# Metabolically versatile psychrotolerant Antarctic bacterium *Pseudomonas* sp. ANT_H12B is an efficient producer of siderophores and accompanying metabolites (SAM) useful for agricultural purposes

**DOI:** 10.1186/s12934-023-02105-2

**Published:** 2023-04-29

**Authors:** M. Musialowski, Ł. Kowalewska, R. Stasiuk, T. Krucoń, K. Debiec-Andrzejewska

**Affiliations:** 1 Department of Geomicrobiology, Institute of Microbiology, Faculty of Biology, University of Warsaw, Miecznikowa 1, 02-096 Warsaw, Poland; 2 Department of Plant Anatomy and Cytology, Institute of Experimental Plant Biology and Biotechnology, Faculty of Biology, University of Warsaw, 02-096 Warsaw, Poland; 3 Department of Environmental Microbiology and Biotechnology, Institute of Microbiology, Faculty of Biology, University of Warsaw, Miecznikowa 1, 02-096 Warsaw, Poland

**Keywords:** Siderophores, Pyoverdine, Psychrotolerant bacteria, Plant-growth promotion, Agriculture, Bacterial metabolites

## Abstract

**Background:**

Bacterial siderophores are chelating compounds with the potential of application in agriculture, due to their plant growth-promoting (PGP) properties, however, high production and purification costs are limiting factors for their wider application. Cost-efficiency of the production could be increased by omitting purification processes, especially since siderophores accompanying metabolites (SAM) often also possess PGP traits. In this study, the metabolism versatility of *Pseudomonas* sp. ANT_H12B was used for the optimization of siderophores production and the potential of these metabolites and SAM was characterized in the context of PGP properties.

**Results:**

The metabolic diversity of ANT_H12B was examined through genomic analysis and phenotype microarrays. The strain was found to be able to use numerous C, N, P, and S sources, which allowed for the design of novel media suitable for efficient production of siderophores in the form of pyoverdine (223.50–512.60 μM). Moreover, depending on the culture medium, the pH of the siderophores and SAM solutions varied from acidic (pH < 5) to alkaline (pH > 8). In a germination test, siderophores and SAM were shown to have a positive effect on plants, with a significant increase in germination percentage observed in beetroot, pea, and tobacco. The PGP potential of SAM was further elucidated through GC/MS analysis, which revealed other compounds with PGP potential, such as indolic acetic acids, organic acids, fatty acids, sugars and alcohols. These compounds not only improved seed germination but could also potentially be beneficial for plant fitness and soil quality.

**Conclusions:**

*Pseudomonas* sp. ANT_H12B was presented as an efficient producer of siderophores and SAM which exhibit PGP potential. It was also shown that omitting downstream processes could not only limit the costs of siderophores production but also improve their agricultural potential.

**Supplementary Information:**

The online version contains supplementary material available at 10.1186/s12934-023-02105-2.

## Background

Siderophores, including those produced by bacteria, are chelating compounds with a high affinity for iron. Their main biological function is to improve the bioavailability and uptake of this crucial element in conditions of iron limitation [1–4]. Bacterial siderophores could also play other biological roles, including non-iron metal transport, toxic metal sequestration, signaling, or protection from oxidative stress. Thus, they have gained the attention of various branches of industry, trying to harness their potential in numerous applications [2, 3, 5, 6]. Bacterial siderophores are considered to be one of the plant-growth-promoting (PGP) agents [2, 7–9]. Their role is to enhance the bioavailability of iron in the soil, which is crucial for proper plant nutrition [2], as well as to stimulate soil microbiota and biocontrol phytopathogens [1, 9–13]. Due to their PGP properties, bacterial siderophores have the potential to be used as soil biostimulants and/or as a component of biofertilizers [14].

Despite the high application potential of bacterial siderophores, many aspects of their production remain inefficient, limiting their broader use. Firstly, the high efficiency of siderophores production is obligatory for industrial-scale production. Although siderophores biosynthesis is common in various microbial taxa, the production rates and efficiency of many strains may be insufficient [9]. Secondly, the optimization of culture conditions for siderophores-producing bacteria (SPB) is highly relevant, including the composition of the medium and pH. The usage of various carbon, nitrogen, and/or phosphorus sources strongly influences the bacterial metabolism profile and affect siderophores’ production efficiency [15–17]. For large-scale applications, a medium for siderophores production should promote a significant yield of these compounds while also being cost-efficient [9]. Another important issue is the cost-inefficient need for heating/cooling during cultivation because bacterial siderophores are usually produced by mesophilic/psychrophilic microorganisms [9, 18, 19]. Finally, the formulation of the siderophores-based product often requires downstream procedure, such as purification (e.g., liquid–liquid or solid-phase extraction, gel filtration, High Performance Liquid Chromatography—HPLC), which make the siderophores production process more complex and expensive [9, 19].

Facilitating and streamlining of siderophores production could be achieved e.g., by the usage of inexpensive substrates for bacteria growth. Since many SPB require the supplementation of medium with expensive amino acids (e.g., l-asparagine) to achieve significant levels of siderophores production, their low-cost production remains a challenging task [9, 19]. Furthermore, the medium for siderophores production has to be nearly completely devoid of iron, which inhibits siderophores biosynthesis [9]. For this reason, the low-cost alternatives of media components, such as waste materials, are mostly unsuitable for siderophores production due to their variable composition and contamination with iron [9, 19]. Therefore, media used for siderophores production should be based on easily accessible and low-cost pure synthetic substrates e.g., inorganic salts [8, 20, 21].

Since metabolites produced by SPB alongside siderophores could possess other PGP traits (e.g., production of phytohormones or organic acids), purification of the final product is not required in the context of agricultural applications, provided there is no phytotoxic effect of siderophores accompanying metabolites (SAM) [9, 14]. Moreover, the concentrations of siderophores obtained in bacterial cultures are environmentally relevant and sufficient for PGP effects [22–24]. Therefore, omitting downstream processes could not only lower the production cost, but also enrich the final product in various plant-beneficial compounds. For this reason SPB bacterial strains, exhibiting complex and diverse metabolism could be great platforms for products with a broad range of PGP properties [14].

In this context, numerous members of *Pseudomonas* genus have the potential to be used for agricultural purposes since they are not only efficient producers of siderophores but can also biosynthesize various secondary metabolites with PGP properties [7, 25, 26]. This metabolic versatility is driven by the diversity of *Pseudomonas*, which due to various physiological and genetic properties are able to thrive in a broad range of environments, including extreme conditions [27–29]. In our previous paper we described *Pseudomonas* sp. ANT_H12B, a psychrotolerant siderophores producer, which exhibited PGP properties with regard to alfalfa (*Medicago sativa* L.) [28]. Due to these characteristics ANT_H12B could potentially be used for the manufacturing of biostimulating agricultural products, however, its biotechnological potential was not fully revealed, especially according to the optimization of siderophores production and the potential PGP role of other accompanying metabolites.

The main goal of the conducted studies was to elucidate the potential of siderophores and accompanying secondary metabolites produced by the psychrotolerant strain *Pseudomonas* sp. ANT_H12B for biostimulation of plant growth. In order to achieve this aim, the following specific goals were accomplished: (i) Genomic and phenotypic analysis of *Pseudomonas* sp. ANT_H12B metabolic potential in order to design cost-efficient media for siderophore production, (ii) the experimental evaluation of siderophores production media, including various carbon and nitrogen sources, (iii) the investigation of the composition of siderophores accompanying metabolites (SAM) produced on various microbial media, as well as (iv) the assessment of the impact of siderophores and SAM on the rate and efficiency of plants germination. The presented studies were performed in the context of the potential application of siderophores produced by *Pseudomonas* sp. ANT_H12B for large-scale agricultural purposes. The knowledge about optimization of the yield and cost production of siderophores based on the example of ANT_H12B strain may be useful for the estimation of the actual biotechnological potential of secondary metabolites produced by representatives of *Pseudomonas* genus.

## Material and methods

### Bacterial strain and plant seeds

The bacterial strain used in this study was *Pseudomonas* sp. ANT_H12B (GenBank assembly accession number: GCA_008369325.1) isolated from the Antarctic soil samples at King George Island (Antarctica; GPS coordinates: 62 09.6010 S, 58 28.4640 W) [29]. Strain exhibits various PGP features, including siderophores biosynthesis (pyoverdine and achromobactin), phosphate solubilization, and indole acetic acid biosynthesis. *Pseudomonas* sp. ANT_H12B genome consists of 6 276 261 base pairs (58.57% GC content) and contains 6168 genes [28].

Untreated plant seeds were used in this study for germination tests. Selected seeds cultivars were characterized by their relevance for agriculture and moderate germination rate/efficiency. The seeds of beetroot (*Beta vulgaris* var. *conditiva* cv. Patryk), pea (*Pisum sativum* L. cv. Iłówiecki) were obtained from Enterprise of Horticulture and Nursery (PNOS), Ożarów Mazowiecki, Poland, and tobacco (*Nicotiana tabacum* L. var, *Xanthi*) was propagated in the in-house growing chambers facilities to obtain sufficient seeds number.

### Bioinformatic analysis

Genomic DNA extraction (cetyl trimethylammonium bromide /lysozyme method), sequencing (Illumina MiSeq platform) and basic genomic analysis (RAST, PATRIC and KEGG services) of *Pseudomonas* sp. ANT_H12B were performed and described in our previous work [28]. In this study additional genomic analysis was used to characterize the genetic background of the phenotypic profile of *Pseudomonas* sp. ANT_H12B and assess the versatility of its metabolism in the context of the siderophores’ production. In the frame of genomic analysis, genes/pathways responsible for carbon metabolism were identified. Bioinformatics analysis of the ANT_H12B genome was performed using MicrobeAnnotator (v.2.0.5) software, and then the KO numbers were mapped to KEGG metabolic pathways and manually curated. The presence of missing enzymes in incomplete metabolic pathways was manually verified using the annotation of the genome deposited in the NCBI database. In addition, a genome search was performed based on the MetaCyc database and available scientific literature.

### Phenotypic profiling of *Pseudomonas* sp. ANT_H12B

Phenotype Microarrays (Biolog Inc., USA) were used to examine the metabolic potential of *Pseudomonas* sp. ANT_H12B. The Phenotype Microarrays (PM) assays involved panels for carbon (PM01 and PM02—190 of C sources), nitrogen (PM03—95 of N sources), as well as phosphorus and sulfur usage (PM04—59 of P and 35 of S sources). PM assays were performed according to the standard protocols recommended by the manufacturer for gram-negative bacteria and described by Gharaie et al. [30]. All assays were performed in triplicates using an OmniLog device (Biolog Inc., USA). All data were collected by OmniLog PM System software (Biolog Inc., USA).

### Optimization of medium chemical composition

#### Bacterial inoculum preparation for siderophores production

To prepare bacterial inoculum for siderophores production, *Pseudomonas* sp. ANT_H12B was cultivated overnight in lysogeny broth (LB) medium at 20 °C with rotary shaking set to 150 rpm. Next, 50 ml of bacterial culture was centrifuged (8000 rpm, 5 min), washed with 0.85% NaCl solution to remove any residues of LB broth, and again centrifuged. This procedure was repeated twice. The final inoculum was prepared by discarding the supernatant and suspending obtained biomass in 50 ml of 0.85% NaCl solution.

#### Selection of media composition and experimental set-up

Based on *Pseudomonas* sp. ANT_H12B phenotypic and genomic profiling, various media designed for siderophores production were prepared. Selected substrates included cost-effective compounds commonly used in industrial/agricultural applications. As a reference medium for siderophores production the GASN medium (7 g L^−1^ glucose, 2 g L^−1^ L-asparagine monohydrate, 0.96 g L^−1^ Na_2_HPO_4_, 0.44 g L^−1^ KH_2_PO_4_, and 0.2 g L^−1^ MgSO_4_ × 7H_2_O) was used [31]. C:N ratio in GASN medium is approximately 7:1. Other media used in this study were designed as modified versions of the GASN medium, using various carbon (glucose, glycerol, ethanol, citric acid) and nitrogen sources (ammonium sulfate, ammonium chloride, ammonium nitrate, l-asparagine), keeping C:N ratio at 7:1 level. The concentrations of phosphates and sulfates in every media were identical to those in the GASN medium. Detailed composition of used media, regarding carbon and nitrogen sources, is presented in Table 1.

**Table 1:** Carbon and nitrogen sources in media used for siderophores production (C:N ratio = 7:1)

Medium	Carbon source (g L^−1^)	Nitrogen source (g L^−1^)
GSA	Glucose 5.25	Ammonium sulfate 1
GCl	Glucose 5.25	Ammonium chloride 1.35
GNO	Glucose 5.25	Ammonium nitrate 1.25
GlSA	Glycerol 4.25	Ammonium sulfate 1
GlASN	Glycerol 4.25	l-asparagine 2
EtSA	Ethanol 5.15	Ammonium sulfate 1
EtASN	Ethanol 5.15	l-asparagine 2
CSA	Citric acid 5.6	Ammonium sulfate 1
CASN	Citric acid 5.6	l-asparagine 2

Media containing various carbon and nitrogen sources (CSA, CASN, EtASN, EtSA, GASN, GCl, GNO, GlASN, GlSA) in seven C:N ratio variants (1:2, 2:1, 3:1, 5:1, 7:1, 10:1, 20:1) were used to examine their influence on siderophores production. Ratio 7:1 (Table 1) was regarded as reference, and other ratios were prepared by increasing or decreasing concentrations of carbon source, while keeping N concentration unchanged. Bacteria were cultivated for 3 days in respective media (pH 7.0) at 10 °C with rotary shaking set to 150 rpm. Initial culture OD_600nm_ was set at 0.06. Conditions were selected according to optimization experiments described in Additional file 1. All experiments were performed in triplicates, using 96-well microplates. The three best variants from every microplate experiment were selected for verification in increased culture volume. For this purpose, bacteria were cultivated for 3 days in conditions identical to the respective microplate assay, with the only difference in the volume of used medium (200 ml). Measurement of microorganisms quantity (CFU ml^−1^), pH and siderophores concentration (CAS assay) were taken every 24 h of the experiment.

### Determination of siderophores productivity of *Pseudomonas* sp. ANT_H12B

To perform a screening estimation of the efficiency of siderophores production in bacterial cultures in various media, CAS (chrome azurol S) reagent was used according to the spectrophotometric method measuring overall siderophores concentration in samples described by Schwyn and Neilands [32]. All measurements were performed in triplicates. The bacterial cultures were centrifuged (8000 rpm for 5 min), and supernatants were added in a 1:1 ratio to the CAS reagent and incubated in darkness for an hour. An automated microplate reader was used to measure the absorbance at 630 nm. A standard curve was obtained using deferoxamine mesylate salt (Sigma-Aldrich), which also served at a concentration of 0.025 mM as a positive control. A sterile medium was used as a negative control.

To determine the concentration of pyoverdine, HPLC analyses were conducted. These analyses were performed for metabolites obtained using selected media: GASN, GCl, GSA, GlSA, GlASN, and CSA after 3 days of cultivation. Bacterial cultures were centrifuged (8000 rpm, 5 min), then supernatants were separated from the biomass and stored in sterile 50 ml tubes in 4 °C for further use. The liquid fractions were transferred to individual tubes and 40 µl of a FeCl_3_ solution (1 M) was added to each tube. The HPLC analyses were carried out using the procedure described by Bultreys et al. [33]. Commercially available pyoverdine from a *Pseudomonas fluorescens* strain (Sigma Aldrich, USA) was used as a standard.

### Chemical analysis of SAM produced by *Pseudomonas* sp. ANT_H12B

The qualitative and semi-quantitative characteristics of SAM were performed with the use of GC–MS analysis. Analyses were performed for metabolites obtained using all studied media after 3 days of cultivation. Bacterial cultures were centrifuged (8000 rpm, 5 min), then supernatants were separated from biomass and stored in sterile 50 ml tubes in 4 °C for further use. All experiments were performed in triplicates.

#### Extraction of organic compounds from the aqueous phase

Organic compounds were extracted from 100 ml of the cell-free aqueous phase of the bacterial cultures and chemical control samples using 25 ml of chloroform in a separatory funnel for 3 min. This procedure was repeated three times. Chloroform extracts were dried with anhydrous Na_2_SO_4_, evaporating the solvent under an N_2_ stream. Samples were then derivatized with 0.5 ml of BSTFA:TMCS, 99:1 (Supelco, USA), for 30 min at 70 °C. A blank sample was prepared according to the same procedure.

#### Analysis of extractable organic compounds—gas chromatography analysis

The separation of organic compounds was performed using an Agilent 7890A Series Gas Chromatograph interfaced with an Agilent 5973c Network Mass Selective Detector and an Agilent 7683 Series Injector (Agilent Technologies, USA). A 5 µl sample was injected with split 1:5 (sample; carrier gas) by 0.3% SD to an HP-5MS column (30 m × 0.25 mm I.D., 0.25 µm film thickness, Agilent Technologies, USA) using He as the carrier gas at 1 ml min^−1^. The ion source was maintained at 250 °C; the GC oven was programmed with a temperature gradient starting at 100 °C (for 3 min), and this was gradually increased to 300 °C (for 2 min) at 6 °C min^−1^. Mass spectrometry analysis was carried out in the electron-impact mode at an ionizing potential of 70 eV. Mass spectra were recorded from m/z 40 to 800 (0–39 min).

#### Selection, identification, and classification of organic compounds

Peaks that indicated an area not less than 0.1% of the total area of the total ion current chromatogram were selected for identification. The identification was performed with an Agilent Technologies Enhanced ChemStation (G1701EA ver. E.02.00.493) and The Wiley Registry of Mass Spectral Data (version 3.2, Copyright 1988–2000 by Palisade Corporation with, 8th Edition with Structures, Copyright 2000 by John Wiley and Sons, Inc.) using a 3% cutoff threshold.

The selected peaks representing organic compounds whose mass spectra indicated compliance with reference mass spectra equal to or higher than 80% were identified. The rest of the organic compounds representing lower compliance (< 80%) were assigned only to the major classes of organic compounds based on the presence of characteristic and dominating fragmentation ions (aromatic hydrocarbons– m/z 65, 77, 78, 79; aliphatic hydrocarbons—m/z 43, 57, 71, 85, 99; alcohols—m/z 45, 59, 73, 87; aldehydes—m/z 44, 58, 72; carboxylic acids—m/z 43, 45, 57, 59, 60, 71, 73, 85, 87). Those organic compounds present in extracts of three repetitions of each sample were selected for further analysis.

### The effect of SAM on seeds germination

The influence of SAM on the rate and efficiency of seeds germination was investigated. For this purpose pea, beetroot, and tobacco seeds were pre-soaked for 30 min in 100 ml of: (i) metabolites solutions produced by *Pseudomonas* sp. ANT_H12B on GASN, GCl, GSA, GlSA, GlASN or CSA medium according to the procedure described in paragraph 4.1, (ii) sterile GASN, GCl, GSA, GlSA, GlASN or CSA medium and (iii) distilled water (control). After 30 min the seeds were drained off, and 25 seeds were placed on a glass petri dish containing lignin soaked with 125 ml of (i) metabolites produced by *Pseudomonas* sp. ANT_H12B on studied media supplemented with Knopp nutrient solution (3 mM Ca(NO_3_)_2_, 1.5 mM KNO_3_, 1.2 mM MgSO_4_, 1.1 mM KH_2_PO_4_, 0.1 mM EDTA—Fe, 5 μM CuSO_4_, 2 μM MnSO_4_ × 5H_2_O, 2 μM ZnSO_4_ × 7H_2_O, 15 nM (NH_4_)_6_Mo_7_O_24_), (ii) sterile GASN, GCl, GSA, GlSA, GlASN or CSA media supplemented with Knopp nutrient solution, (iii) Knopp nutrient solution. To ensure a comparable level of nutrients in obtained metabolites and initial media, the amount of carbon source was reduced by half in the sterile media variant, according to experimentally estimated usage during siderophores production. Each variant was performed in four repetitions. The seeds were incubated in the dark for 7 days at 20° C. Every day the number of germinating seeds was counted. Germination percentage (GP) was calculated using the following equation [34]:

$$GP\left[\%\right]=\frac{total \,number\, of \,seeds \,germinated}{total \,number \,of \,seeds \,per \,petri\, dish} \times 100$$

### Statistical analysis

Statistical analysis was performed using RStudio 2022.02.2 software [35]. One-way analysis of variance (ANOVA) at p ≤ 0.05 was used to test the significance of the differences in groups during optimization and germination experiments. To test pairwise differences in groups Tukey Honestly Significant Difference (HSD) tests were used at p ≤ 0.05. The results were presented on graphs obtained with ggplot2 v3.3.5 [36].

## Results

### Analysis of genomic potential to use various carbon sources

Genomic analysis of *Pseudomonas* sp. ANT_H12B performed in our previous studies showed that this strain can obtain energy by metabolizing carbohydrates through glycolysis, oxidative and non-oxidative phases of the pentose phosphate cycle, Entner-Doudoroff glucose catabolism, d-galactonate degradation, and glycogen degradation, as well as beta-oxidation of fatty acids and degradation of acylglycerols [28]. In order to estimate the full metabolic potential of the strain showcased in phenotype profiling, in-depth genomic analysis was carried out to identify genetic determinants associated with carbon conversion pathways. The obtained data indicated the ability to degrade various compounds e.g., (i) monosaccharides (e.g., xylose transformed by xylose isomerase to d-xylulose and in the next steps to ribulose-5-phosphate, which is intermediate of pentose phosphate pathway) and disaccharides (d-trehalose transformed by trehalase to glucose), (ii) alcohol sugars (d-mannitol transformed by mannitol 2-dehydrogenase to d-fructose and glycerol transformed by glycerol kinase into glycerone-phosphate. Both products could be involved into glycolysis), (iii) amino sugars (n-acetyl-d-glucosamine transformed by n-acetylglucosamine PTS system to N-acetylglucosamine-6-phosphate and in the three next steps to d-fructose-6-phosphate, which is glycolysis intermediate), (iv) alcohols (dihydroxyacetone transformed by multi phosphoryl transferase to glycerone-phosphate) and (v) carboxylic acids (including fumaric acid and acetic acid transformed to acetyl-CoA respectively by fumarate hydratase in the first step and malate synthase in second step, and acetate-CoA ligase). Genomic analysis also revealed the remarkable ability to use amino acids as a carbon source, including the majority of proteinogenic amino acids (70.00%). l-Alanine and l-Serine could be transformed into pyruvate (by alanine-synthesizing transaminase and l-serine ammonia-lyase, respectively) and eventually enter the Krebs cycle. Most other proteinogenic amino acids used as C-source could enter the Krebs cycle transformed into its intermediates: oxoglutarate, oxaloacetate or Acetyl-CoA. Moreover, several dipeptidases were identified in *Pseudomonas* sp. ANT_H12B. A list of enzymes identified for carbon compounds was included in the Additional file 2.

### Phenotype profile of *Pseudomonas* sp. ANT_H12B regarding the using of various C, N, S, P sources

**Fig. 1:**
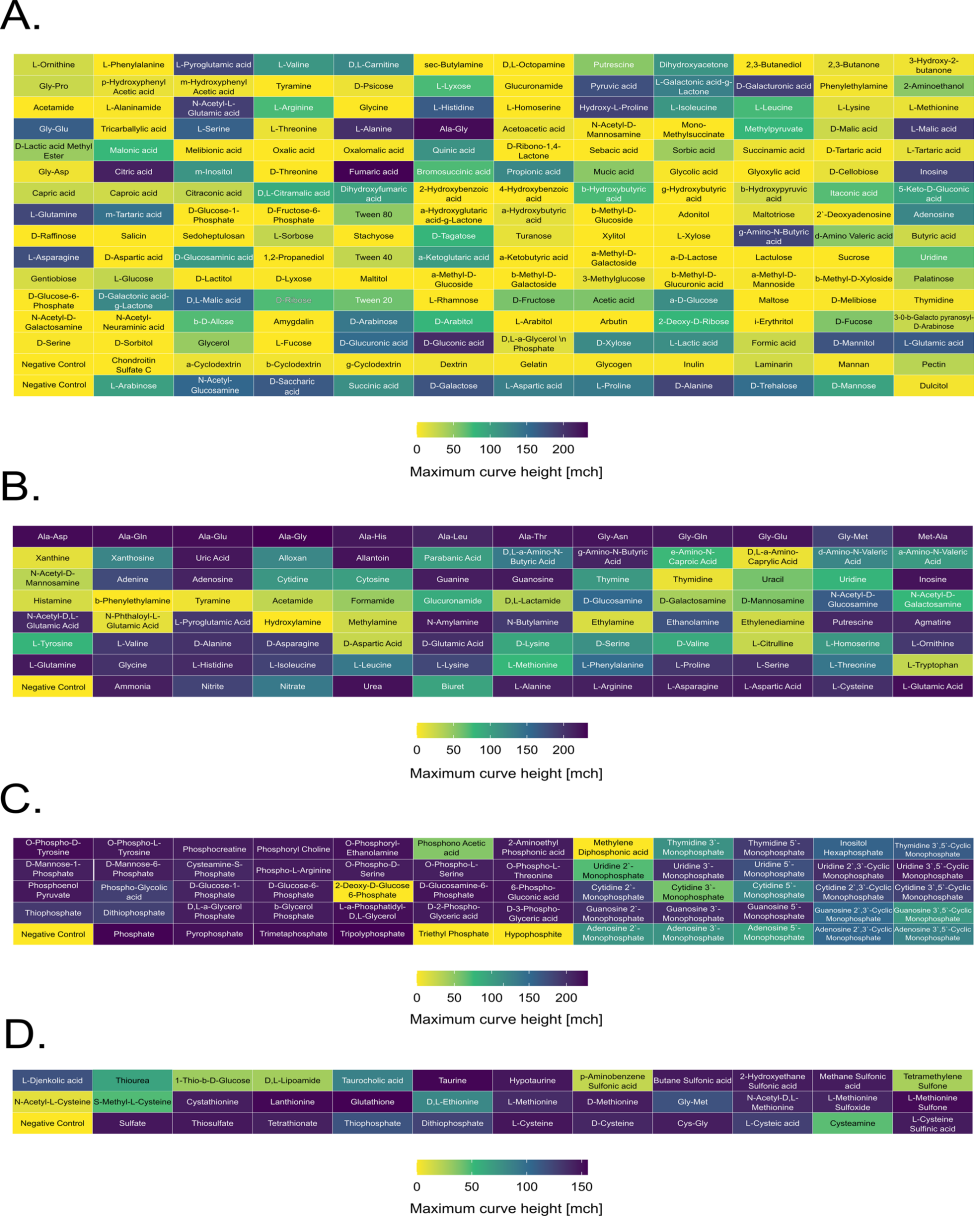
Results of BIOLOG phenotypic profiling assay of *Pseudomonas* sp. ANT_H12B for carbon source (**A**), nitrogen source (**B**), phosphorus source (**C**) and sulfur source (**D**). Color scale shows the maximum curve height parameter (mch)

During a PM assay, a wide array of 395 compounds was tested as a source of essential nutrients for bacterial growth (Fig. 1). We estimated growth on each compound using the parameter of maximum curve height (mch) [30]. The threshold level for positive growth result was calculated separately for all carbon (PM01 and PM02), nitrogen (PM03), phosphorus, and sulfur sources (PM04). For each nutrient source the positive threshold was set at 5.00% of the maximal value of mch obtained in given experiment. Obtained results showed the broad metabolic potential of *Pseudomonas* sp. ANT_H12B, which exhibited growth using 52.11% of carbon, 94.74% of nitrogen, 93.22% of phosphorus, and 100.00% of sulfur sources tested in this study.

Among all tested nutrients, the use of carbon by *Pseudomonas* sp. ANT_H12B was the most selective (Fig. 1A). Positive growth signals were observed in 99 of 190 tested carbon sources. Particular compound groups were more preferential for bacterial growth than others. The strain was able to grow using all tested fatty acids and esters as a C source. It also showed the ability for the use of the majority of tested d-monosaccharides (83.33%), amino acids (70.00%), carboxylic acids (65.00%), nucleosides (60.00%) and l-monosaccharides (57.15%). ANT_H12B was less adapted to use amino sugars (33.33%), alcohol sugars (33.33%), trisaccharides (33.33%), disaccharides (30.00%), amines (20.00%), polymers (18.00%). The growth of the strain was not observed in the presence of amides. *Pseudomonas* sp. ANT_H12B was able to metabolize the vast majority of examined nitrogen sources (Fig. 1B). Growth was exhibited in 90 of 95 tested compounds (94.74%), belonging to various chemical groups, including peptides (100.00%), proteinogenic L-amino acids (100.00%), proteinogenic D-amino acids (100.00%), non-proteinogenic amino acids (100.00%), amino sugars (100.00%), nucleosides (83.33%), amines (81.81%) and inorganic compounds (75.00%).

The phenotypic assay showed the versatility of phosphorus (Fig. 1C) and sulfur (Fig. 1D) metabolism in *Pseudomonas* sp. ANT_H12B. All 30 tested sulfur sources were suitable for bacteria growth, with no distinctive difference in growth rate between organic or inorganic compounds. Regarding phosphorus, the strain used 55 of 59 tested P-sources. Positive results were observed in all nucleoside phosphates samples, 90.00% other organic and 85.71% inorganic compounds.

### Optimization of medium composition for siderophores production—screening tests

**Fig. 2:**
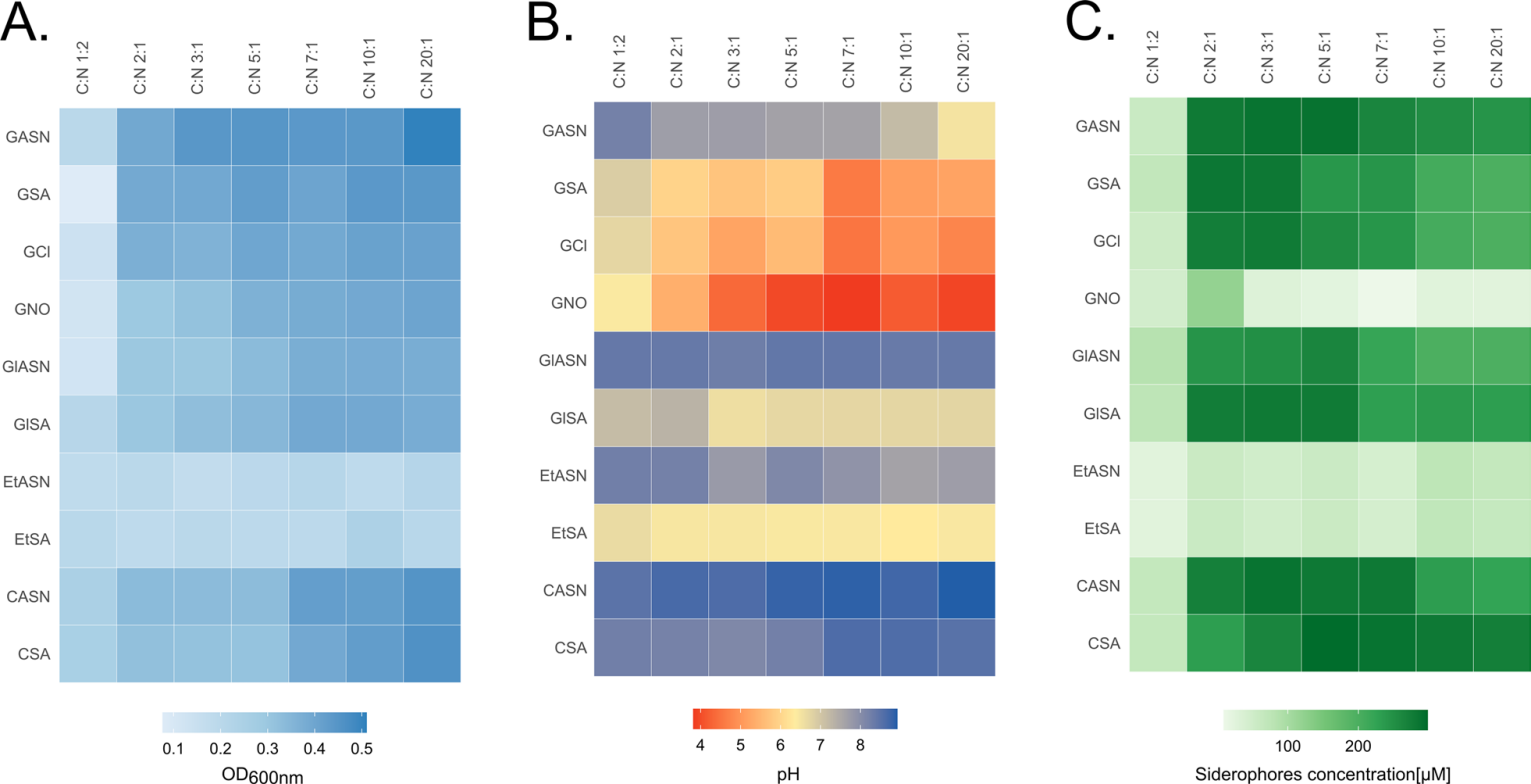
Optimization of medium composition. Results for tested media (GASN, GSA, GCl, GNO, GlSA, GlASN, EtSA, EtASN, CASN, CSA) in seven C:N ratio: **A** culture density (OD_600nm_ at the end of cultivation), **B** pH (measured at the end of cultivation), and **C** the highest measured siderophores concentration

*Pseudomonas* sp. ANT_H12B was able to grow using the majority of tested carbon (glucose, glycerol, citric acid) and nitrogen sources (l-asparagine, ammonium sulfate, ammonium chloride, ammonium nitrate) (Fig. 2A). Only the use of ethanol as a carbon source resulted in inhibited growth. C:N ratio significantly influenced observed bacterial growth (ANOVA test F = 64.52, p-value = 2.14 × 10^–9^). Pairwise statistical analysis showed no significant differences between 20:1, 10:1, 7:1, and 5:1 ratios, in which the most intense bacterial growth was observed.

Media composition strongly influenced the pH of the culture (Fig. 2B). Inorganic nitrogen sources (ammonium sulfate, chloride, and nitrate) combined with glucose, glycerol, and ethanol resulted in the acidification of the media. The lowest pH was observed with the use of glucose, reaching the level of 4–5 after 3 days. pH in media with glycerol and ethanol was slightly higher, and after 3 days, it reached 6–6.5. The magnitude of acidification increased with a rising C:N ratio. l-asparagine as a nitrogen source or citric acid as a carbon source were alkalization factors. The highest pH (> 8) was observed when citric acid (CASN, CSA) and glycerol (GLASN) were used as carbon sources. In these media, pH was not significantly affected by the change in the C:N ratio. The alkalization rate was lower with the use of glucose (GASN) and ethanol (EtSA), and the pH level dropped to neutral (EtSA) or even slightly acidic (GASN) with the increase in the C:N ratio.

Siderophores production over 200 μM was observed with the use of the majority of tested carbon (glucose, glycerol, citric acid) and nitrogen (l-asparagine, ammonium sulfate, ammonium chloride) sources (Fig. 2C). Only in media with ethanol as a carbon source and ammonium nitrate as a nitrogen source, significantly lower siderophores concentrations (below 100 μM) were observed compared to other tested variants. Therefore, media EtSA, EtASN, and GNO were excluded from further testing. C:N ratio was also an important factor influencing significantly siderophores production (ANOVA test F = 10.43, p-value = 4.52 × 10^–10^). Three most optimal C:N ratios for siderophores production were selected using pairwise statistical tests: 2:1, 3:1, and 5:1 for GASN, GSA, GCl, GlSA, and GlASN media, 3:1, 5:1 and 7:1 for ClASN medium and 5:1, 7:1 and 10:1 for CSA medium. For every tested medium 1:2, C:N ratio was the least efficient variant, where siderophores production was strongly inhibited and fell under 100 μM.

### Optimization of medium composition for siderophores production—verification tests

Five media (GCl, GSA, GlSA, GlASN, CSA) that promoted the highest siderophore production in optimization tests, and GASN medium as a reference, were selected for verification tests in larger volume. Based on optimization test results, three optimal variants of the C:N ratio were chosen for every tested medium. Significant differences were observed between media in siderophores production after 3 days (ANOVA test F = 63.12, p-value = 4.44 × 10^–9^). Pairwise analysis revealed that the highest siderophores biosynthesis was associated with GSA and GCl (average 503.00 μM and 496.00 μM, respectively). The second group included media with lesser efficiency of siderophores production, which were GASN, GlASN, and GlSA (average 431.00 μM, 407.00 μM, and 401.00 μM, respectively). The least amount of siderophores was produced with use of CSA (average 329.00 μM). Among media containing glucose (GASN, GCl, GSA), significantly higher siderophores production was observed with the use of C:N ratios of 3:1 and 5:1. In other media C:N ratio did not affect siderophores production. According to these results, variants of media allowing for maximal production of siderophores with the lowest C:N ratios (GASN 3:1, GSA 3:1, GCl 3:1, GlSA 2:1, GlASN 2:1, and CSA 5:1) were selected for further tests.

### The effect of medium composition on the pyoverdine concentration

**Fig. 3:**
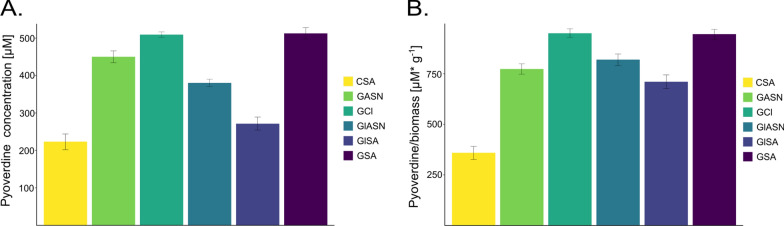
HPLC analysis of pyoverdine content in tested media. Results shown for concentration in bacterial culture **A** and pyoverdine/biomass ratio (**B**)

Metabolites produced on selected media were analyzed using HPLC to estimate the exact concentration of pyoverdine in each sample. The results showed similar patterns to the CAS assay with 512.60 μM concentration in GSA, 509.30 μM in GCl, 450.20 μM in GASN, 380.40 μM in GlASN, 271.40 μM in GlSA and 223.50 μM in CSA medium. Pyoverdine production was also calculated as the yield of product in terms of biomass (μM siderophores g^−1^ biomass): 950.19 in GCl, 945.76 in GSA, 819.83 in GlASN, 773.54 in GASN, 710.47 in GlSA and 359.32 in CSA (Fig. 3).

### Chemical characteristic of SAM produced by *Pseudomonas* sp. ANT_H12B

The GC/MS analyses were performed to identify SAM with potential plant growth-promoting traits. We selected producing media according to the results of optimization experiments (GSA 3:1, GCl 3:1, GlASN 2:1, GlSA 2:1, CSA 5:1, and GASN 3:1 as the reference). Among detected compounds, fatty acids, other organic acids, alcohols, esters, indolic acid and its derivatives, sugars, nitrogen-containing organic compounds, sulfur-containing organic compounds, hydrocarbons, and aromatic compounds were observed (Table 2). A semi-quantitative estimate of the total concentration of each group was made and the specific species of each compound identified under the other classification were described (with a minimum probability match of 70%). Fatty acids were the dominant group of compounds in metabolites obtained from every media, except CSA, with concentrations ranging from 40.48 mg L^−1^ (CSA) to 225.96 mg L^−1^ (GASN). The majority of detected fatty acids were long-chained (14–18 carbon atoms). Both unsaturated (9-octadecenoic acid, cis-13-octadecenoic acid, cis-9-hexadecenoic acid, cis-9-Octadecenoic acid, trans-13-octadecenoic acid, trans-9-octadecenoic acid) and saturated (3-hydroxymyristic acid, 3-trimethylsiloxymyristic acid, n-pentadecanoic acid, hexadecenoic acid, tetradecanoic acid, octadecanoic acid) fatty acids were observed. The most abundant long-chain fatty acids, detected in all samples, were hexadecenoic acid, cis-9-hexadecanoic acid, and dodecanoic acid. Medium-chain fatty acids were also identified (6–12 carbon atoms), including saturated (3-hydroxydecanoic acid, dodecanoic acid, hexanoic acid, nonanoic acid) and unsaturated (2-octenoic acid) compounds.

**Table 2:** Chemical class/concentrations of selected metabolites produced by *Pseudomonas* sp. ANT_H12B, based on GC/MS analysis

Compound group	GASN (mg L^−1^)	GSA (mg L^−1^)	GCl (mg L^−1^)	GlASN (mg L^−1^)	GlSA (mg L^−1^)	CSA (mg L^−1^)
Fatty acids	225.962	73.805	80.131	111.739	87.669	40.484
Octadecanoic acid	0	1.587	3.222	2.964	1.347	2.140
n-pentadecanoic acid	0.633	0	1.000	0.216	0	1.408
Tetradecanoic acid	0	1.143	4.486	5.453	1.210	0
Cis-9-hexadecenoic acid	68.088	29.467	26.643	46.549	28.473	6.868
Hexadecanoic acid	56.505	20.511	28.690	35.575	37.322	2.892
Cis-9-Octadecenoic acid	0	0	2.553	1.811	0	3.167
Trans-13-octadecenoic acid	0	15.683	7.864	11.914	14.185	0
3-Hydroxydecanoic acid	9.582	0.609	0	0.830	0.561	7.880
Dodecanoic acid	25.418	0.430	1.266	2.357	0.735	7.002
Nonanoic acid	0.991	0	2.132	0.797	1.538	0
Octadecanoic acid	0	1.587	3.222	2.964	1.347	2.140
Organic acid	48.954	4.143	0	8.372	2.509	118.902
1,2-benzenedicarboxylic acid	16.108	4.143	0	8.372	2.509	0
Sugars	6.959	4.241	0.170	0	0	0
Indolic acid	0.02632	0.00076	0.00252	0.01251	0.00543	0.00269
Alcohols	0	0	7.686	2.540	5.875	0
Esters	4.069	2.676	7.580	0.035	10.977	9.167
Organic sulfur	0	4.202	3.016	4.979	0	13.931
Organic nitrogen	11.843	1.784	30.413	1.083	12.396	0

In metabolites obtained from CSA medium dominant group of compounds were organic acids other than fatty acids (118.90 mg L^−1^). However, mainly due to the presence of 1,2,3-propanetricarboxylate. Organic acids other than fatty acids were also detected in significant amounts in metabolites from GASN medium (48.95 mg L^−1^) and in much smaller quantities in GlASN (8.37 mg L^−1^), GSA (4.14 mg L^−1^), and GlSA (2.51 mg L^−1^).

Indoleacetic acid (IAA) and its derivatives were identified in all tested media. The highest concentration of IAA was measured in GASN (0.026 mg L^−1^) and GlASN (0.013 mg L^−1^) medium. In media containing inorganic nitrogen source, IAA concentrations were lower (GSA- 0.0075 mg L^−1^, GCl—0.0025 mg L^−1^, GlSA—0.0054 mg L^−1^, CSA—0.0026 mg L^−1^).

### The effect of SAM on seed germination

**Fig. 4:**
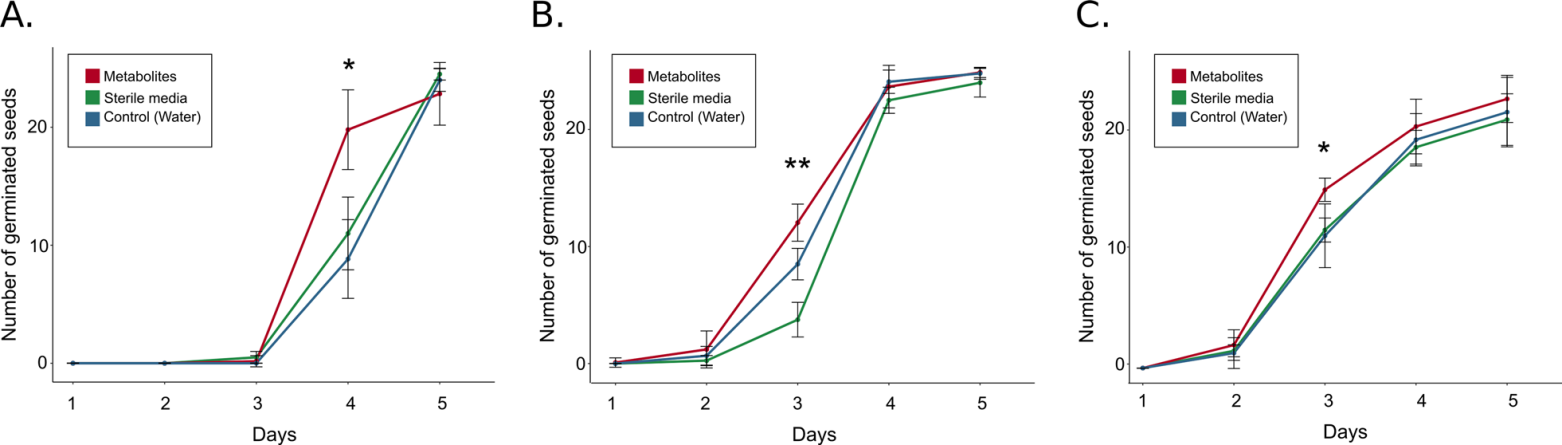
Germination test results for **A** peas, **B** beetroot, and **C** tobacco. Significant differences between experimental groups in every day were estimated using ANOVA test and are represented by asterisk above respective group in given day (***− p < 0.001, **− p ≤ 0.01, * − p ≤ 0.05, no asterisk − p > 0.05)

Seed germination tests were performed to reveal the effect of SAM on plant (beetroot, pea, and tobacco) growth. Media for metabolites production were selected based on the best results obtained from optimization experiments regarding siderophores production. Seeds germination percentage (GP) was similar among all tested variants for the first two days in beetroot and pea and three days in tobacco. On the third day of the experiment in pea treated with metabolites GP was higher than in control and sterile media variants (33.16% and 14,16% respectively) and the number of germinated seeds was significantly higher (ANOVA test F = 12.35, p-value = 0.013, Fig. 4A). For metabolites treated beetroot also on the 3rd day GP was 11.00% higher than control, 14.16% higher than in sterile media, and the number of germinated seeds was significantly higher (ANOVA test F = 24.74, p-value = 0.0025; Fig. 4B). A similar effect was observed for tobacco on day 4, with GP higher in metabolites treated seeds than in control and sterile media variants (35.16% and 43.83% respectively) and the number of germinated seeds was significantly higher (ANOVA test F = 7.16, p-value = 0.028, Fig. 4C).

## Discussion

### Phenotype profile of *Pseudomonas *sp. ANT_H12B

Results of genomic and phenotypic analysis showed the metabolic versatility of *Pseudomonas* sp. ANT_H12B. We identified various enzymes involved in the metabolism of organic compounds, which provide the ability to use them as a carbon source in main pathways of energy metabolism. Many of these organic compounds are particularly abundant in the Antarctic environment, thus the ability to use them is an important adaptation of *Pseudomonas* sp. ANT_H12B to survive in harsh regions. For example, trehalose, mannitol, and glycerol are frequently found in Antarctic soil because, as a compatible solute, one of their profound biological roles is osmo- and cryo-protection, especially important in cold environments [37–39]. These compounds could be used as a C source by ANT_H12B due to the presence of genes encoding e.g., trehalase, mannitol 2-dehydrogenase, and glycerol kinase.

We confirmed the genomic-based hypothesis of *Pseudomonas* sp. ANT_H12B metabolic versatility during PM tests. This strain exhibited great metabolic flexibility, which could be regarded as outstanding among other members of the genus *Pseudomonas*. *Pseudomonas* sp. ANT_H12B was able to use 52.10% of tested carbon sources. Other members of the genus *Pseudomonas* obtained lower results during their respective tests, including environmental strains e.g., *Pseudomonas putida* from vineyard soils was able to use 30.50% of C sources [40], eight *Pseudomonas* strains isolated from rhizosphere were able to use 18.10–23.60% of C sources [41] and clinical isolate *Pseudomonas stutzeri,* was able to use 26.80% of C sources [42]*. Pseudomonas* sp*.* ANT_H12B shared with other *Pseudomonas* strains the ability for the efficiently use organic acids, while exceeding their capability of metabolizing carbohydrates and amino acids. The carbon source usage of *Pseudomonas* sp. ANT_H12B was also remarkable compared to other soil microorganisms*,* including *Rhodococcus* (37.90–38.95% of C sources) [43], *Rhizobium* (35.80%) [44] and *Sinorhizobium meliloti* (40.00%) [45]. *Pseudomonas* sp. ANT_H12B was also able to use a vast majority of nitrogen sources (94.70%), which exceeded the ability of *Pseudomonas stuzeri* (77.90%) [42], *Rhizobium* (approximately 54.30%) [44], *Rhodococcus* (approximately 65.30%) and *Sinorhizobium meliloti* strains (approximately 88.00%) [45].

We demonstrated the ability of *Pseudomonas* sp. ANT_H12B to use peptides and amino acids both as a carbon and nitrogen source, confirming results obtained during genomic analysis, in which we identified many genes encoding enzymes involved in amino acid and protein metabolism. Such ability is characteristic of many psychrotolerant bacteria since the main nitrogen input to soil in polar environments is in the form of proteins or short peptides, which decomposition is slower due to low temperatures [46]. Short peptides are one of the biggest contributors of the soil-dissolved nitrogen pool of polar environments. Microbial communities can directly take them up and subject them to further decomposition inside the cells [46–48]. *Pseudomonas* sp. ANT_H12B exhibited adaptation to these conditions possessing several dipeptidases and enzymes that allow for further transformation of amino acids to main metabolic pathways intermediates and use them as C and N sources.

### The efficiency of siderophores production by *Pseudomonas* sp. ANT_H12B

Bacterial siderophores production varies significantly. Some bacteria can biosynthesize in culture media approximately 10 M of siderophores (*Azotoacter vinelandii)* [49], while others exhibit a production rate of 1.6 mM (*Streptomyces olivaceus*) [50]. However, the concentration of siderophores in culture media usually ranges between 100 and 200 µM [9]. This diversity is driven by various factors e.g., culture conditions, medium composition, and bacterial taxonomy [9]. Significant differences can also be observed in closely related microorganisms, even in the same species, e.g., three different strains of *Azotobacter vinelandii* producing siderophores in concentrations of 10 [49], 140 [51], or 360 µM [51]. Members of the *Pseudomonas* genus are described as efficient producers of greenish-pigmented siderophores – pyoverdine [52]. However, the diversity of pyoverdine production is also observed within this taxon. Moderate pyoverdine producers can obtain concentrations of 25-80 µM [53], while more efficient strains described in the literature are able to obtain 260-300 µM [54, 55]. In this context, *Pseudomonas* sp. ANT_H12B producing as high as 510 μM, can be regarded as a very efficient bacterial siderophores producer, with its outstanding pyoverdine biosynthesis rate among other *Pseudomonas* bacteria. Moreover, we performed detailed HPLC analysis to confirm qualitatively and quantitatively pyoverdine production. Unfortunately, in many studies, pyoverdine concentration is estimated only using CAS assay, which is valuable as a screening method, but it lacks precision in siderophores quantification since chelating compounds other than siderophores could affect its results [56].

Temperature is one of the most critical factors influencing bacterial culture dynamics, and it also significantly impacts siderophores' production efficiency. It has been reported in several studies that the optimal temperature for siderophores production is often similar to optimal or sub-optimal for bacterial growth [9]. The majority of described siderophores producers are mesophilic bacteria with a preference for moderate temperatures in the range of 25–37 °C [9, 57, 58]. Although many psychrotolerant or psychrophilic microorganisms has been described as siderophores producers, the specific data about their productivity and characteristics are scarce and describe this process only in a qualitative approach. In our study, we characterized siderophores production in low temperatures in more detail, including biotechnological aspects of culture conditions and a quantitative approach. Results showed that *Pseudomonas* sp. ANT_H12B, an example of a psychrotolerant microorganism, exhibits very efficient pyoverdine production in a broad range of temperatures (4–22 °C). This flexibility could benefit biotechnological use since extensive temperature control is not required [9].

The composition of the growth medium, particularly the carbon and nitrogen sources, plays a crucial role in siderophores production [59]. Carbon, being a major component of biomass, significantly affects genetic and physiological processes, leading to varied qualitative and quantitative composition of produced metabolites [15–17]*.* In the case of siderophores production, many microorganisms exhibit a preference for gluconeogenic substrates (organic acids), especially those from the *Pseudomonas* genus [9, 16]. It has been proposed that gluconeogenic substrates increase metabolic fluxes toward the Krebs Cycle, providing an increased supply of pyoverdine intermediates [16]. However, contrary to those observations, *Pseudomonas* sp. ANT_H12B exhibited the highest siderophores production when glycolytic substrate (glucose) was used. This finding suggests that the metabolic profiles of psychrotolerant bacteria can differ significantly, even in microorganisms from the similar taxon [60, 61]. *Pseudomonas* strains generally prefer organic acids due to the regulation of catabolic repression and absence of phosphofructokinase, an important enzyme in the glycolytic pathway [25]. However, in the genome of *Pseudomonas* sp. ANT_H12B, we identified phosphofructokinase gene, indicating that carbon metabolism in this strain differs from that described in most *Pseudomonas* strains from moderate climates. Further studies of *Pseudomonas* sp. ANT_H12B metabolism, physiology, and genetics could reveal more about the specifics of psychrotolerant soil microorganisms.

Nitrogen sources in the medium did not strongly influence siderophores production by *Pseudomonas* sp. ANT_H12B. Several studies have shown that adding amino acids as a nitrogen source could improve siderophores production in *Pseudomonas* strain, e.g., L-asparagine [31] or glutamic acid [62]. In the case of *Pseudomonas* sp. ANT_H12B, both organic and inorganic nitrogen sources, resulted in efficient siderophores production.

The efficiency of siderophores production has been also linked with the pH of the culture. The decrease of medium pH has been shown to correlate with a reduction of siderophores concentration in media, as they are labile in acidic environments [9, 59, 63]. Higher pyoverdine biosynthesis was associated with neutral to slightly alkaline conditions [9, 19, 63]. During our study we showed a different pattern of pyoverdine production by *Pseudomonas* sp. ANT_H12B, which was not inhibited by the low medium pH. Moreover, we obtained the highest rate of pyoverdine production, confirmed by HPLC analysis, using GCl or GSA medium, which resulted in significant acidification of culture conditions.

### Plant growth-promoting properties of siderophores and SAM

Pyoverdine production could be regarded as the most important PGP activity of *Pseduomonas* sp. ANT_H12B, due to efficiency of this process and its high plant-stimulating potential. Pyoverdine is described as one of the most important siderophores in agricultural context [2, 7, 8, 14, 52, 64]. Pyoverdine could significantly improve plant nutrition since Fe-Pyoverdine complexes are providing iron to various plants more efficiently than Fe-EDTA complexes [22, 23]. In field experiments with pea (*Pisum sativum*), pyoverdine improved plant supply not only with iron, but also with other nutrients (e.g., Zn or Mg) [23]. Moreover, pyoverdine efficiently provides iron for plants with various Fe-uptake strategies [22, 23, 65]. However, this effect could be observed in iron-limited soil conditions.

In our study we elucidated the role of other metabolites produced during biosynthesis of pyoverdine. Overall effect of SAM was tested by using them as a priming agent for pea, tobacco, and beetroot seeds in germination tests. Results showed not only SAM lack of phytotoxicity, but also stimulation of seeds germination percentage. Positive role of various metabolites was shown also in other seed germination experiments e.g., priming of triticale (*Triticale hexaploide* L.) seeds with melatonin resulted in increased germination rate by 57.67% [66]. In other experiments it was shown that treatment of seeds with gibberellic acid and/or Indole Acetic Acid (IAA) improved germination parameters and subsequent cultivation of Masson pine (*Pinus massoniana*) and *Aspilia Africana* [67, 68]. PGP potential of bacterial metabolite was studied in germination tests of pepper and maize, where treatment with cell free supernatant from *Bacillus* sp. AS19 significantly improved the process [24].

Among chemical compounds identified in SAM was IAA, which could have a major role in improvement of seed germination. IAA is a plant hormone belonging to the auxin class, which has been linked with the regulation of various plant physiological processes, including growth and development. The main biological roles of IAA include the regulation of cell division, elongation, and differentiation [69–71]. We observed biosynthesis of IAA with use of every tested medium, obtaining the best results using L-asparagine-containing media GASN and GlASN. The addition of amino acids was shown in other papers as a factor potentially increasing the bacterial biosynthesis of IAA [72–74]. Exogenous bacterial IAA in the rhizosphere positively impacts plant growth, mainly through root development stimulation, which improves nutrient uptake [75, 76]. Moreover, foliar application of IAA resulted in increased leaf area and plant height [77, 78]. Application of IAA in the concentration of 0.017 mg L^−1^(similar to concentrations obtained in SAM composition) was also demonstrated as a seed germination promoter in experiments with Masson pine (*Pinus massoniana*) [68].

PGP potential of SAM could be broader than direct stimulation of seed germination, due to presence of plant-beneficial compounds, which activity could be exhibited in soil conditions e.g., organic acids (OA), which we also identified in SAM. This group of compounds is important in sustainable agriculture since it is associated with various PGP properties [79]. OA could directly improve plant fitness, due to involvement in nutrient acquisition, e.g., by solubilization and mobilization of micronutrients and macronutrients [79–82]. Phosphorus and potassium are vital plant macronutrients and are present in the soil in large amounts. However, they mainly exist in a form of insoluble minerals and their bioavailability is limited [79]. It was shown that various bacterial carboxylic acids, e.g., propionic acid, lactic acid, 2-ketogluconic acid, citric acid, tartaric acid, acetic acid, oxalic acid, glycolic acid, succinic acid, inalonic acid, and fumaric acid were involved in solubilization of P and K in soils [79, 80, 82, 83]. OA could also improve the soil bioavailable zinc pool, which deficiency is one of the most widespread among plants [79, 81].

In GC/MS analysis, we showed that the majority of compounds found in SAM produced by *Pseudomonas* sp. ANT_H12B cultures belong to the subgroup of organic acid, specifically fatty acids. These compounds are very important in cold adaptation processes of bacteria [84–86]. Bacteria growing in low-temperature conditions change their membrane chemical composition, increasing the amount of unsaturated fatty acids (UFA) [84, 85, 87]. A higher UFA content enables the cell to maintain increased membrane fluidity, which is crucial at lower temperatures and prevents membrane destabilization [84, 85, 87, 88]. The application of fatty acids in agriculture could be beneficial, as the antifungal activities of these compounds was described. Antagonistic activities of various fatty acids (butyric acid, caproic acid, caprylic acid, capric acid, lauric acid, myristic acid, palmitic acid, oleic acid, and linoleic acid) was shown against phytopathogenic fungi [89]. In other studies, bacteria from *Bacillus* and *Pseudomonas* genes inhibited the mycelial growth of *Fusarium solani* through the emission of volatile compounds, including fatty acids [90]. The biocontrol properties of fatty acids could be also useful against eukaryotic parasites, as antagonistic activity of nine compounds from this group was shown against nematode *Meloidogyne incognita*, which is responsible for root infections [91].

SAM could also increase overall soil quality and fertility in an indirect mode of action, by stimulating soil microorganisms, which eventually could improve plant health and growth. Several studies have shown that application of OA to soil is associated with increased diversity of microbial communities, positively affecting PGP microorganism [79, 92, 93]. Nitrogen-fixing bacteria (NFB), which reduce atmospheric nitrogen into ammonia, are an important PGP group of microorganisms that contribute to soil fertility by increasing the availability of this crucial element [94]. Many NFBs preferentially use OA as a carbon source; thus their numbers and activity could be increased by addition of OA to soil [95]. Additionally, other groups of compounds found in SAM, e.g. sugars and alcohols, could also be beneficial for microbial growth and contribute to soil microbiome quality [96, 97].

One of the most important properties of SAM was their variable pH, which ranged from 4.5 to 8.5, depending on medium composition. Despite this variability, we were able to identify compounds with PGP properties e.g., siderophores, auxins or organic acids in every studied SAM. pH is one of the most important factors that affect soil properties, both abiotic (e.g. nutrients availability) or biotic (microbiome composition and activity) [98, 99]. Therefore, different plant species have varying optimal soil pH ranges, from acidic to alkaline. In this context, variability of SAM pH could be advantageous in agriculture since it allows for the development of tailored products to meet the specific needs of different crops [19, 99].

## Conclusions

With the use of genomic and phenotypic analysis, we optimized the siderophores production process by developing five novel media compositions using various carbon and nitrogen sources, which allow for improving production cost-efficiency. Metabolites produced with each medium shared a high concentration of pyoverdine but varied significantly in pH, enabling their use in different soil and plant context. In particular, we identified a high concentration of pyoverdine in acidic media (pH < 5), which is unique in siderophores research, as these compounds typically degrade under low pH conditions. We also demonstrated that during the production of siderophores on each newly designed medium, other PGP compounds were produced, e.g., auxins, organic acids, and fatty acids and we showed their growth-stimulating potential in germination tests of pea, beetroot, and tobacco seeds. Our findings indicated that unpurified siderophores solutions containing accompanying PGP metabolites—SAM, could be the basis for plant-stimulating bioproducts since they not only reduce production costs but also provide the added value of various PGP compounds. In our study we highlighted the importance of using metabolically versatile bacteria, such as *Pseudomonas* sp. ANT_H12B, to harness the full PGP potential of microbes for agriculture.

## Supplementary Information

**Additional file 1.** Optimization of physicochemical and biological conditions for efficient siderophores production.

**Additional file 2.** List of enzymes involved in carbon metabolism identified in Pseudomonas sp. ANT_H12B genome.

## Data Availability

The datasets used and/or analyzed during the current study are available from the corresponding author upon reasonable request.
