# Supplementary material for: Metabolically versatile psychrotolerant Antarctic bacterium Pseudomonas sp. ANT_H12B is an efficient producer of siderophores and accompanying metabolites (SAM) useful for agricultural purposes
Source: Microb Cell Fact. 2023 Apr 29;22:85. doi: 10.1186/s12934-023-02105-2 (PMC10149013; doi:10.1186/s12934-023-02105-2)
Supplement: Supplementary file 1 — Additional file 1. Optimization of physicochemical and biological conditions for efficient siderophores production. [file 12934_2023_2105_MOESM1_ESM.doc]

Supplementary Materials – SM_1

*Optimization of physicochemical and biological conditions for efficient siderophores production*

To estimate the optimal culture conditions for siderophores production by *Pseudomonas* sp. ANT_H12B the influence of various physicochemical and biological factors on bacterial growth and metabolites production efficiency was investigated.

Examination of optimal conditions for siderophores production was performed using a GASN medium (7 g L^-1^ glucose, 2 g L^-1^ L-asparagine monohydrate, 0.96 g L^-1^ Na_2_HPO_4_, 0.44 g L^-1^ KH_2_PO_4_, and 0.2 g L^-1^ MgSO_4_ x 7H_2_O) [36], inoculated with previously prepared inoculum with initial OD_600nm_ 0.06. Then, bacteria were cultivated for 3 days at 10° C with rotary shaking set to 150 rpm. All experiments were performed in triplicates in 96-well microplates in working volume of 200 μl. Experimental layout was randomized and border microplate cells was filled only with sterile medium (control). Particular culture conditions were altered in specific experimental variants: temperature of culture (4, 10, 15, 22, 30, and 37° C), initial OD_600nm_ (0.01, 0.03, 0.06, 0.08, 0.1, 0.15, 0.2, 0.3 ) and shaking speed (0, 100, 150, 200, 250 rpm). Measurement of optical density in a microplate reader (Sunrise TECAN, Tecan Trading AG, Männedorf, Switzerland), pH (Mettler Toledo FiveEasy PLUS FP20 with InLab Micro probe), and siderophore concentration (CAS assay) were taken every 24 hours of the experiment. From every screening test experiment, three best variants were selected and verified in a increased volume scale. For this purpose, bacteria were cultivated for 3 days in conditions identical to the respective screening assay, with the only difference in the used volume of the medium, which was 200 ml. Measurement of microorganism's quantity (CFU/ml), pH and siderophores concentration (CAS assay) were taken every 24 hours during the experiment.

*Shaking speed*

Screening analyses performed in various shaking speeds from 0 to 250 rpm on microplates have revealed that this factor also significantly influenced siderophore production efficiency (ANOVA test F= 253.5, p-value= 5.29 x 10^-10^). Moderate shaking speed (150-200 rpm) was the most beneficial for siderophore production, resulting in a concentration range of 246-260μM (Figure S1A). Bacterial growth was constrained with low (0 and 100 rpm) or high (250 rpm) shaking intensity, which resulted in a concentration of siderophores under 200μM. Verification tests were performed with 100, 150, and 200 rpm. Obtained results confirmed that the highest siderophore production was associated with shaking speeds of 150 rpm and 200 rpm (433μM and 428μM, respectively). 150 rpm speed was selected for further experiments.

*Initial inoculation*

Initial inoculum quantity also influenced siderophore production in microplate tests (ANOVA test F= 240.3, p-value= 5.14 x 10^-15^). However, in every variant from OD_600nm_ 0.06 to 0.3, siderophore concentration was comparable after 3 days of cultivation (in the range of 273-291μM), and Tukey HSD pairwise test confirmed the lack of significant differences between those variants (Figure S1B). Only in the cases of initial OD_600nm_ 0.01 and 0.03 significantly decreased siderophores production was observed. Initial inoculum quantities of OD_600nm_ 0.06, 0.08 and 0.1 were selected for flask verification tests. No statistically significant differences were observed (ANOVA test F= 0.068, p-value= 0.935). Thus initial inoculum quantity of OD_600nm_ 0.06 was selected for further experiments as sufficient for obtaining the best efficiency of the process, with the utilization of minimal inoculum quantity.

*Cultivation temperature*

Cultivation temperature significantly influenced siderophore production efficiency during microplate tests (ANOVA test F= 1028, p-value= 2,34 x 10^-15^). *Pseudomonas* sp. ANT_H12B exhibited growth and siderophores production in a temperature range of 4-22° C. In higher temperatures, growth was significantly inhibited, and no siderophores were detected (Figure S1C). Optimal conditions for siderophores production included 10° C and 15° C variants, in which significantly higher siderophore production (276μM and 272μM, respectively) than in other variants, confirmed in Tukey HSD pairwise analysis. At 4° C and 22° C , siderophore production was about 20-30% lower. The temperature did not affect the pH of the culture, and in every variant, alkalization of medium was observed. Aiming for the most effective siderophore production in possibly lowest temperature, variants 4° C , 10° C , and 15° C were chosen for verification tests. During all experiments performed in lab flasks, siderophore concentration was generally about 40 - 50% higher, than in respective variants in microplates. The highest siderophore concentration was confirmed for 10° C and 15° C (426μM and 421μM, respectively). Temperature 10° C was selected as the optimal culture temperature due to the lower energy consumption than in 15° C.


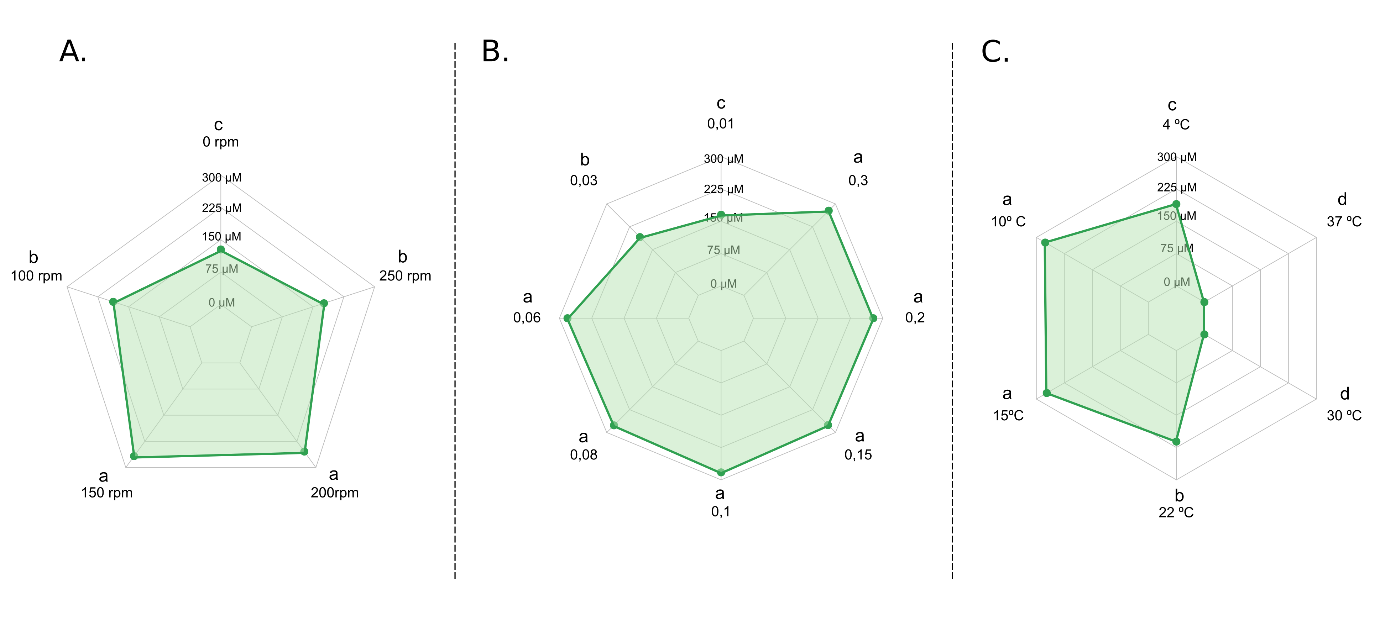
*Figure S1: The influence of physicochemical and biological conditions on efficiency of siderophore production in microplate optimization experiments: (A) shaking speed, (B) Initial inoculation and (C) temperature. Each axis represents maximal obtained siderophore concentration using given parameters.* *Significant differences between variants were obtained by Tukey HSD test and indicated on figure by letters above the bars (if two measurements do not share a letter, they are statistically different with p ≤ 0.05)*

*Response Surface Analysis*

To confirm selection of optimal conditions for siderophore production Response Surface Analysis of verification tests results were performed with use of R package rsm. Every tested parameter consisted of three levels: as a “0” level was selected the optimal variant selected in verification tests, and neighboring levels were selected as “high” and “low” (In summary selected levels: shaking speed - 100, 150, and 200 rpm, OD – 0.03, 0.06 and 0.08 and 4° C, 10° C, 15° C). Measured response was siderophore production. Second order model was calculated with rsm function SO(), Obtained model allowed for identification of optimal experimental points for siderophore production, which included temperature – 9.97° C, shaking speed 157.84 rpm and OD - 0.055 (Figure S2).


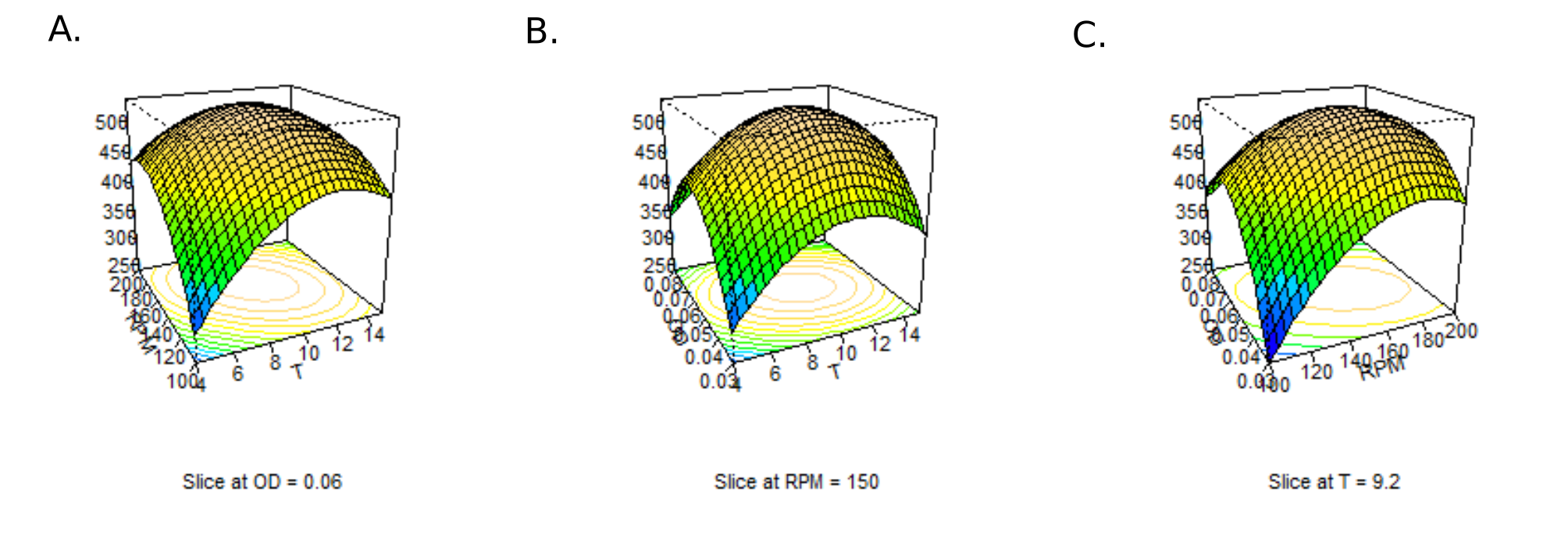


*Figure S2: Graphical representation of Response Surface Model obtained with rsm R package and fixed value of (A) OD = 0.06, (B) shaking speed = 150 rpm and (C) temperature = 9.2. Model’s adjusted R-square = 0.9883 and lack-of-fit has 0 degree of freedom.*
